# Physician Burnout and Work Satisfaction in the Cardiac Intensive Care Unit: Sponsored by the Critical Care Cardiology Section of the American College of Cardiology

**DOI:** 10.1016/j.jacadv.2026.102629

**Published:** 2026-03-25

**Authors:** Samuel B. Brusca, Alexander I. Papolos, Michael A. Solomon, Robert O. Roswell, Rosy Thacil, Benjamin Kenigsberg, Sunit-Preet Chaudhry, Eugene Yuriditsky, Ran Lee, Alejandra Gutierrez, Jacob C. Jentzer, Christopher F. Barnett

**Affiliations:** a Division of Cardiology, Department of Internal Medicine, University of California-San Francisco, San Francisco, California, USA; b Division of Cardiology, Department of Critical Care Medicine, MedStar Washington Hospital Center, Washington, District of Columbia, USA; c Critical Care Medicine Department, National Institutes of Health Clinical Center, Bethesda, Maryland, USA; d Cardiovascular Branch, National Heart Lung and Blood Institute, National Institutes of Health, Bethesda, Maryland, USA; e Department of Cardiology, Lenox Hill Hospital, Northwell Health, The Zucker School of Medicine at Hofstra/Northwell, New Hyde Park, New York, USA; f Department of Cardiology, Elmhurst Hospital/Mount Sinai College of Medicine, New York, New York, USA; g Ascension St. Vincent Heart Center, Indianapolis, Indiana and Ascension St. Vincent Cardiovascular Research Institute, Indianapolis, Indiana, USA; h Division of Cardiology, Department of Medicine, NYU Grossman School of Medicine, New York, New York, USA; i Sections of Critical Care and Heart Failure Transplant Cardiology, Department of Cardiovascular Medicine, Cleveland Clinic, Cleveland, Ohio, USA; j Department of Cardiovascular Medicine, University of Minnesota, Minneapolis, Minnesota, USA; k Department of Cardiovascular Medicine, Mayo Clinic, Rochester, Minnesota, USA

**Keywords:** burnout, cardiac intensive care unit, critical care, survey, work satisfaction

## Abstract

**Background:**

Physician burnout represents a modern medical crisis, especially among high-intensity specialties.

**Objectives:**

This is the first study to investigate physician burnout in the cardiac intensive care unit (CICU).

**Methods:**

A 42-item survey was administered to U.S. and Canadian CICU-focused physicians, designed in part to assess burnout and work satisfaction.

**Results:**

Of 1,085 contacted physicians, 289 completed the survey for a response rate of 27%; 218 (75%) of these were eligible for analysis, including 207 from the U.S. and 11 from Canada. Most respondents were White (62%) males (70%), aged 31 to 50 years (61%). Approximately 1/3 of physicians were American Board of Internal Medicine dually certified in cardiology and critical care medicine. Seventy percent reported being very satisfied with their current job, although less than half (48%) were happy with their work-life balance or required professional work outside the hospital (41%). Overall, 35% of respondents reported some degree of burnout, with higher rates among women compared to men (53% vs 30%, *P* = 0.03) and in later career stages (≥8 years vs <8 years; 43% vs 25%, *P* = 0.04). Burnout was not associated with number of day or nighttime shifts, nor salary or type of board certification. One-third of respondents were planning to leave their current job or reduce clinical hours, predominantly due to burnout (61%).

**Conclusions:**

Burnout is more common in women and later career stages and is the leading reason cited by CICU-focused physicians that are considering practice change. It affects 1 of 3 CICU-focused physicians and impacts work satisfaction.

Physician burnout is increasingly recognized as an international crisis that affects physician well-being, both patient satisfaction and outcomes, and results in increased heath care system costs.^1^, ^2^, ^3^ The concept of burnout was described in the 1970s as the emotional exhaustion, depersonalization, and detachment developed by professionals in intense, service-oriented work environments.^4^ The term has been widely adopted by both popular media and in the scientific literature with accepted research metrics measuring feelings of emotional exhaustion, depersonalization, and a depressed sense of accomplishment.^5^ Not surprisingly, burnout is widely reported among the physician workforce, with prevalence rates of 30% to 50% often identified. Contributing factors include long work hours, administrative responsibilities, and perceived lack of decision-making autonomy.^4^^,^^6^^,^^7^ Awareness and national attention on burnout among physicians was notably fostered by the COVID-19 pandemic, with reported peak rates of burnout (>60%) in 2021 and recent reductions in the years following the height of the virus’s impact.^8^^,^^9^

Critical care cardiology (CCC) represents a developing subspecialty within cardiology, which has largely arisen in response to the increased complexity of cardiac intensive care unit (ICU) (CICU) patients, including older age, prevalence of multiorgan failure, and utilization of advanced mechanical, cardiac, and respiratory support.^10^ There is a noted shortage of American Board of Internal Medicine dually-certified CCC physicians, with fewer than 160 dually certified in both cardiology and critical care medicine in the United States as of 2023. There are additional critical care cardiologists practicing via the legacy pathway while only being certified in cardiovascular medicine or cardiovascular medicine and an additional cardiovascular disease subspecialty (eg, interventional cardiology).^10^, ^11^, ^12^ Critical care cardiologists represent a unique ICU workforce with heterogeneous job responsibilities/clinical workloads often overlapping with noncardiology critical care medicine physicians that have a cardiac-focused practice in mixed specialty units and surgical CICU settings. CICU-focused physicians (physicians with a substantial CICU practice focus, including cardiologists and noncardiologists) are frequently exposed to emotionally demanding situations, including cardiac arrests and end of life care, and their daily clinical workload is often unpredictable. Furthermore, CICU-focused physicians typically work long hours, including a significant burden of clinically active overnight in-hospital and home call. Thus, exploration of burnout among CICU-focused physicians is vital to the development of burnout mitigation strategies and the survival/growth of the CCC subspecialty. To this end, The American College of Cardiology’s (ACC) CCC member section created a workforce survey that included analyses of physician burnout and job satisfaction.^13^ We hypothesized that burnout would be common among CICU-focused physicians and that it would be more prevalent in those with more demanding ICU schedules, including night coverage.

## Methods

Members of the ACC’s CCC section created a 42-item online, multiple-choice survey, which was distributed to U.S. and Canadian physicians, including critical care cardiologists and noncardiology CICU-focused physicians. The full survey is available in the Supplement. Survey questions included demographics as well as queries regarding job satisfaction and burnout. Advarra Institutional Review Board Services reviewed the study protocol and using the Department of Health and Human Services regulations found at 45 CFR 46.104(d), determined that this research was exempt from Institutional Review Board oversight.

The survey link was emailed to 1,085 physicians, identified either by the ACC database as being critical care cardiologists or as outreach from members of the ACC CCC section. Respondents were unable to complete the survey if they reported no longer being in practice, spending no time rounding as the primary attending in a medical or surgical CICU, or if they were not board certified or board eligible in any field of medicine. Respondents that identified as physicians in training that had not yet accepted attending job offers were also excluded; however, physicians in training that had accepted jobs were able to complete the survey, to capture their negotiated schedules, clinical duties, salaries, and model of CICU care delivery. The survey was open from October 25, 2023 to March 4, 2024. The data were collated as counts and percentages for categorical variables and median values with 25% to 75% IQR for continuous variables. Quality of life/job satisfaction was assessed using a 5-point Likert scale with 1 representing not at all satisfied and 5 representing extremely satisfied. The burnout assessment was adapted from the mini-Z survey with the following potential responses: 1) I enjoy my work. I have no symptoms of burnout; 2) occasionally I am under stress, and I don’t always have as much energy as I once did, but I don’t feel burned out; 3) I am definitely burning out and have one or more symptoms of burnout, such as physical or emotional exhaustion; 4) the symptoms of burnout I’m experiencing won’t go away. I think about frustration at work a lot; and 5) I feel completely burned out and often wonder if I can go on. I am at the point where I may need some changes or may need to seek some sort of help.^14^ Physicians choosing 1 or 2 were considered without burnout and physicians choosing 3 through 5 were considered positive for burnout as previously described.^15^^,^^16^

Comparisons among physicians with and without burnout were made using chi-square analysis and across burnout responses using one-way analysis of variance. Correlations between burnout and job satisfaction were assessed with 2-tailed spearman rho. Statistical analyses were performed in SPSS (version 27, IBM), with a 2-tailed *P* value significance cutoff of *P* ≤ 0.05. As this was an exploratory analysis, adjustment for multiple comparisons was not performed. A subgroup analysis was performed on only those physicians responding that reported being either board eligible or certified in cardiovascular medicine (ie, CICU-focused cardiologists) and excluding physicians in training that had accepted job offers.

## Results

### Study sample

Of the 1,085 physicians that received the survey, 289 responded for a response rate of 27%. Seventy-one respondents encountered hard stops while answering the survey leading to termination of their responses and exclusion from analysis. Reasons for exclusion included (multiple selections possible): physicians not board certified in any medical specialty (n = 3), physicians in training not having accepted an attending position (n = 39), physicians no longer in practice (n = 6), and physicians not rounding as the primary attending in any CICU (n = 40). After excluding these 71 respondents, 218 participants were left in the analysis sample.

### Characteristics of respondents

The majority of the 218 physicians in the analysis sample were White (n = 135, 62%), males (n = 152, 70%), aged 31 to 50 years old (n = 133, 61%). A total of 170 (78%) of respondents were CICU-focused cardiologists, with fewer of the CICU-focused physicians specializing in alternative fields, such as critical care medicine, pulmonary medicine, or anesthesia (Table 1). Notably, approximately one-third (n = 79, 36%) of responding physicians were American Board of Internal Medicine dually-certified critical care cardiologists, which accounts for approximately half of all such dually-certified providers in the United States.

**Table 1:** CICU-Focused Physician Demographics (N = 218)

Sex	
Male	152 (69.7%)
Female	39 (17.9%)
Not reported	27 (12.4%)
Age	
31-40 years old	61 (28.0%)
41-50 years old	72 (33.0%)
51-60 yrs old	26 (11.9%)
61-70 years old	26 (11.9%)
71 years and older	9 (4.1%)
Not reported	24 (11.0%)
Career stage	
In training	4 (1.8%)
1-7 years	79 (36.2%)
8-14 years	44 (20.2%)
15-21 years	21 (9.6%)
22 or more years	42 (19.3%)
Not reported	28 (12.8%)
Board certification (multiple selections possible)	
Cardiovascular disease	170 (78.0%)
Internal medicine	127 (58.3%)
Critical care medicine	114 (52.3%)
Echocardiography	72 (33.0%)
Advanced HF/transplant cardiology	38 (17.4%)
Interventional cardiology	29 (13.3%)
Anesthesia	19 (8.7%)
Neurocritical care	9 (4.1%)
Pulmonary medicine	6 (2.8%)
Electrophysiology	4 (1.8%)
Emergency medicine	3 (1.4%)
Surgery	2 (0.9%)
Other	22 (10.1%)
Cardiovascular disease and critical care medicine	79 (36.2%)
Employment status	
Currently in training, but have accepted a position	4 (1.8%)
Employed part-time	6 (2.8%)
Employed full-time	204 (93.6%)
Other	4 (1.8%)
Practice affiliation	
University affiliated academic medical center	145 (66.5%)
Nonuniversity associated academic medical center	37 (17.0%)
Community hospital	27 (12.4%)
VA or other government hospital	12 (3.2%)
Other, please specify	2 (0.9%)
Academic appointment/faculty rank	
Instructor	5 (2.3%)
Assistant professor	97 (44.5%)
Associate professor	44 (20.2%)
Professor	34 (15.6%)
Other	1 (0.5%)
None, do not have an academic appointment	34 (15.3%)
No answer	3 (1.4%)
Race/ethnicity	
White	135 (61.9%)
Black/African American	9 (4.1%)
Hispanic/Latin	19 (8.7%)
Asian	44 (20.2%)
Native American/Alaskan Native	0 (0.0%)
Native Hawaiian/Pacific Islander	0 (0.0%)
Other	6 (2.8%)
Do not wish to disclose	16 (7.3%)
Cardiac transplant and MCS capabilities of institution	
Perform heart transplant	141 (64.7%)
Implant and manage durable LVAD	156 (71.6%)
Initiate and manage V-A ECMO	182 (83.5%)
Initiate and manage MAFP	194 (89.0%)
Initiate and manage IABP	204 (93.6%)

CICU = cardiac intensive care unit; HF = heart failure; IABP = intra-aortic balloon pump; LVAD = left ventricular assist device; MAFP = microaxial flow pump; MCS = mechanical circulatory support; VA = Veterans Affairs; V-A ECMO = veno-arterial extracorporeal membrane oxygenation.

Most physicians had been in practice for 7 years or less (n = 79, 36%) with an associated academic appointment of assistant professor (n = 97, 45%). Only 4 respondents were physicians in training that had accepted job offers with the overwhelming majority being employed full time (n = 204, 94%) at university affiliated (n = 145, 67%) or nonuniversity affiliated (n = 37, 17%) academic medical centers. These centers were predominantly level 1 CICU centers with the ability to initiate and manage intra-aortic balloon pump (94%), transvalvular microaxial flow pump (89%), and venoarterial extracorporeal membrane oxygenation (84%).^10^ Fewer centers offered durable left ventricular assist device surgery (72%) and orthotopic heart transplantation surgery (65%) (Table 1).

### Job satisfaction

**Figure 1:**
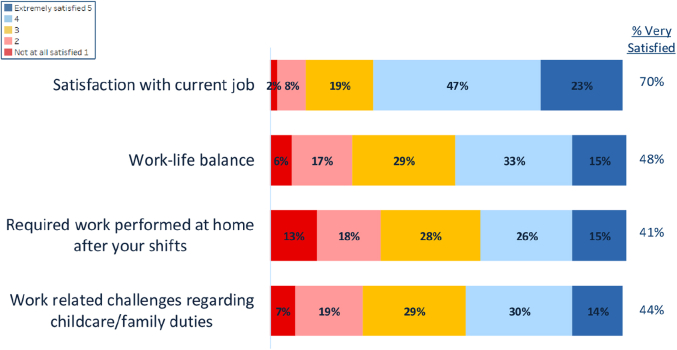
**Cardiac Intensive Care Unit–Focused Physician Job Satisfaction** Survey responses of CICU-focused physicians describing their satisfaction with their current job, work-life balance, at-home work, and balance between work and family duties. Respondents rated their satisfaction from 1 (not at all satisfied, dark red) to 5 (extremely satisfied, dark blue) with scores of 4 and 5 considered “very satisfied.” Nonrespondents were excluded leading to a variable sample size for each question, ranging from n = 187 to 212.

CICU-focused physicians were asked to rate their satisfaction with their current job overall, work-life balance, necessary after-hours work, and work-related challenges due to family duties on a 1 to 5 scale with 5 being extremely satisfied and 1 being not at all satisfied (Figure 1). Seventy percent of respondents reported being very satisfied with their current job (rating 4 or 5) with 19% responding neutrally (rating 3) and 10% reporting dissatisfaction (rating 1 or 2). Fewer physicians were satisfied with their work-life balance, with only 48% responding favorably and 29% neutrally. Similarly, a minority (41%) of respondents were satisfied with required work at home as well as the impact work has on family duties (44%) (Figure 1).

### Burnout

**Figure 2:**
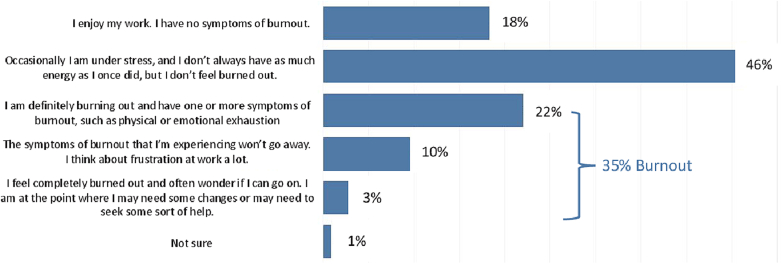
**Cardiac Intensive Care Unit–Focused Physician Burnout Survey Responses From 215 Cardiac Intensive Care Unit–Focused Physicians Describing Their Degree of Burnout** Physicians responding that they are “definitely burning out,” that their symptoms of burnout “won’t go away,” or that they “feel completely burned out,” were characterized as having burnout. Physicians responding that they have no symptoms of burnout or that they are under stress but “don’t feel burned out,” were characterized as not having burnout. Thirty-five percent of respondents had burnout, whereas 64% had no burnout. Only 1% responded that they were not sure.

**Figure 3:**
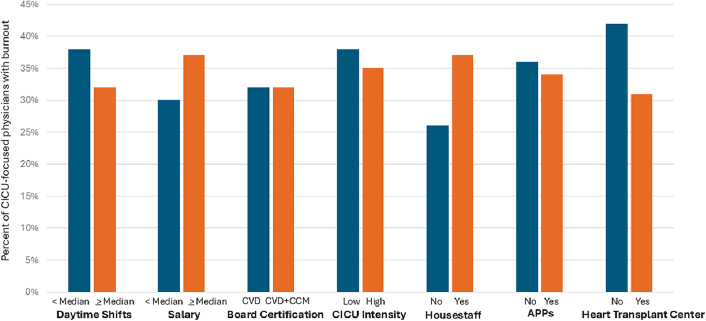
**Burnout Comparisons Among CICU-Focused Physicians** Rates of burnout were compared among CICU-focused physicians based on demographic data as well as their responses to other survey questions regarding CICU job responsibilities and care models. There were nonsignificant trends toward increased burnout in physicians working in CICUs with trainees (36.9% vs 25.6%; *P* = 0.18) and in physicians at centers not performing heart transplant surgery (42.1% vs 30.9%; *P* = 0.10). No differences in burnout were present based on CICU staffing intensity (with high intensity indicating critical care medicine or critical care cardiology primary/co-management and low-intensity indicating that any credentialed cardiologist can manage their patient in the CICU) or the presence of advanced practice providers. There were also no differences in burnout based on the number of total annual daytime shifts, salary, or comparing those with cardiology training only vs dually certified providers in cardiology and critical care medicine. APP = advanced practice provider; CCM = critical care medicine; CICU = cardiac intensive care unit; CVD = cardiovascular disease.

CICU-focused physicians were asked to describe whether they were suffering from burnout, with 35% affirming experiencing a significant burden of burnout (Table 2, Figure 2, Central Illustration). Only 18% of respondents reported no burnout at all. The presence of burnout was negatively correlated with job satisfaction (ρ = −0.582, *P* < 0.01) as well as work-life balance (ρ = −0.568, *P* < 0.01). Burnout was more common in women than in men (53% vs 30%; *P* = 0.03) as well as those in later career stages (43% vs 25%; *P* = 0.04) (Table 2, Central Illustration). Nonsignificant trends toward increased burnout were identified in CICU-focused physicians who were older than 50 years (41.7% vs 31.3%; *P* = 0.36), those working with housestaff in the CICU (36.9% vs 25.6%; *P* = 0.18), and those working at centers not performing heart transplantation (42.1% vs 30.9%; *P* = 0.1) (Table 2, Figure 3). There were no differences in burnout among CICU-focused physicians working with advanced practice providers or those working in high intensity as compared to traditional/low-intensity CICUs (Table 2, Figure 3).

**Table 2:** CICU-Focused Physician Burnout as Relates to Individual and CICU Demographics

	N	No Burnout	Burnout	*P* Value^b^
	215^a^	65.1%	34.9%	
Sex				0.03
Men	150	70.0%	30.0%	
Women	38	47.4%	52.6%	
Not reported	27	63.0%	37.0%	
Years in practice				0.04
1-7 y	81	75.3%	24.7%	
8 or more years	106	57.5%	42.5%	
Not reported	28	64.3%	35.7%	
Age				0.36
31-50 y old	131	68.7%	31.3%	
51 y and older	60	58.3%	41.7%	
Not reported	24	62.5%	37.5%	
Medical and surgical CICU beds				0.37
1 to 20	32	59.4%	40.6%	
More than 20	145	68.3%	31.7%	
Not reported	38	57.9%	42.1%	
CICU staffing intensity^c^				0.59
Traditional/low-intensity	63	61.9%	38.1%	
High-intensity	113	64.6%	35.4%	
Not reported	39	71.8%	28.2%	
Housestaff in the CICU				0.18
No	39	74.4%	25.6%	
Yes	176	63.1%	36.9%	
APPs in the CICU				0.82
No	61	63.9%	36.1%	
Yes	154	65.6%	34.4%	
Heart transplants performed				0.1
No	76	57.9%	42.1%	
Yes	139	69.1%	30.9%	

APP = advanced practice provider; other abbreviation as in Table 1.

a 3 respondents did not answer burnout questions.

b Chi-square testing.

c High-intensity defined as involvement as critical care medicine or critical care cardiology as primary or comanaging providers; Traditional/low-intensity defined as a CICU in which any credentialed cardiologist can manage patient’s they admit to the CICU.

**Central Illustration:**
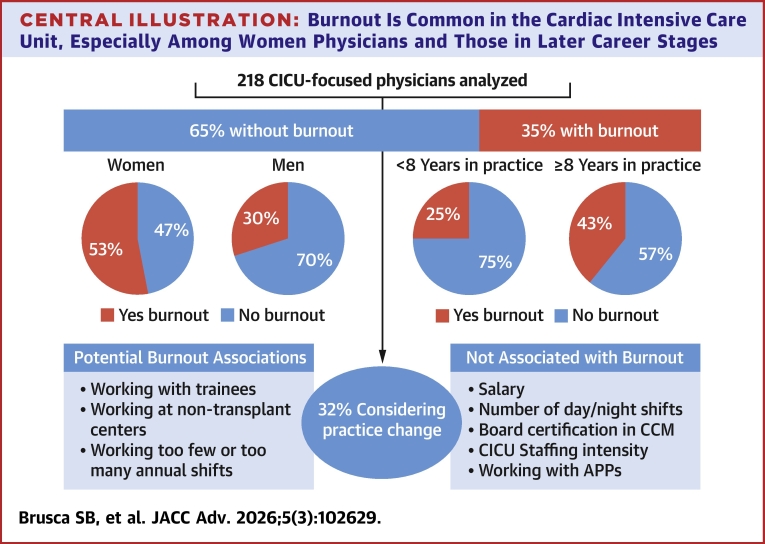
Burnout Is Common in the Cardiac Intensive Care Unit, Especially Among Women Physicians and Those in Later Career Stages Of the 289 physicians responding to the survey, 218 CICU-focused physicians were eligible for inclusion in analysis. Of these providers, approximately one-third reported symptoms of burnout and were considering career changes. Burnout was more common in women compared to men (52.6% vs 30%; *P* = 0.03) as well as in mid-late career physicians (42.5% vs 24.7%; *P* = 0.04). There were no clear associations between burnout and physician salary, number of day, night, or home-call shifts, or related to type of board certification, intensity of CICU staffing (with high intensity indicating critical care medicine or critical care cardiology primary/co-management and low-intensity indicating that any credentialed cardiologist can manage their patient in the CICU), or the presence of APPs in the CICU. Hypothesis generating trends were identified, potentially indicating a relationship between burnout and working at non-quaternary centers (not offering heart transplant), working with housestaff, and working too few or too many total annual ICU shifts. CICU = cardiac intensive care unit; APP = advanced practice provider; CCM = critical care medicine.

No differences in the rate of burnout were identified between those physicians above vs below the median number of daytime shifts (60), in-house nighttime shifts (0), or home-call night shifts (30) (Table 3, Figure 3). There were also no differences in burnout when comparing those above vs below the median salary ($375,000), those with fewer or more days free of clinical duty following a CICU week of service, or those dually certified in cardiology and critical care medicine as compared to physicians certified in cardiology only or other specialty combinations (eg, cardiovascular disease and echocardiography (Table 4, Figure 3). Finally, additional exploratory analyses were performed, analyzing burnout as relates to the above variables as quartiles instead of comparing above/below the median. Burnout trends remained largely similar in the quartile analysis (Supplement Table 1), with trends toward increased burnout in older CICU-focused physicians at later career stages. In addition, a potential relationship between total annual ICU shifts and burnout emerged, with those working 55 to 110 annual shifts suffering significantly less burnout (19.6%) than those working fewer (0-54 annual shifts; 45.3% burnout) or more (111-169 annual shifts; 40.4% burnout & >169 annual shifts; 35.2% burnout; *P* = 0.03).

**Table 3:** CICU-Focused Physician Burnout as Relates to Number of ICU Shifts

	N	No Burnout	Burnout	*P* Value^a^
Daytime Shifts				0.32
Below median (60)	99	61.6%	38.4%	
Median (60) or above	116	68.1%	31.9%	
In-house overnight shifts				0.81
Median (0)	141	64.5%	35.5%	
Above median (0)	74	66.2%	33.8%	
At home overnight shifts				0.22
Below median (30)	108	61.1%	38.9%	
Median (30) or above	107	69.2%	30.8%	

Abbreviation as Table 1.

a Chi-square testing.

**Table 4:** CICU-Focused Physician Burnout as Relates to Salary, Days Free of Service, and Board Certification

	N	No Burnout	Burnout	*P* Value^a^
Current salary				0.57
Below median (375,000)	76	69.7%	30.3%	
Median (375,000) or above	118	62.7%	37.3%	
Not reported	21	61.9%	38.1%	
Days free of clinical duty^b^				0.69
0-2 d	122	63.9%	36.1%	
3 or more d	53	69.8%	30.2%	
No reported	18	72.2%	27.8%	
Board certification				0.69
Dual critical care/cardiology	78	67.9%	32.1%	
Cardiology only	31	67.7%	32.3%	
Other specialty combinations	106	62.3%	37.7%	

Abbreviation as in Table 1.

a Chi-square.

b Days free of clinical duty following a 7-day CICU block.

**Figure 4:**
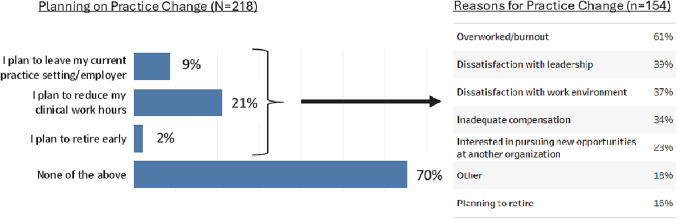
**Cardiac Intensive Care Unit–Focused Physician Intent to Change Practice** Of 218 surveyed CICU-focused physicians, 33% reported an intent to change practice, indicating a plan to change clinical setting/employer (9%), to reduce work hours (21%), or to retire early (2%). Of these, 154 went on to list the reasons for potential practice change, with burnout (61%), dissatisfaction with leadership (39%), dissatisfaction with work environment (37%), and inadequate compensation (34%) being most commonly cited.

Mirroring the burnout rate, 32% of responding physicians described an intent to change practice, including transitioning to a new clinical setting, reduced clinical hours, or early retirement (Figure 4). Of the physicians who went on to describe their reasons for practice change (n = 154), most cited burnout (61%), dissatisfaction with leadership (39%) and work environment (37%), or inadequate compensation (34%).

A subgroup analysis was performed only including survey respondents (n = 166) that were board eligible/certified in cardiovascular medicine and excluding physicians in training that had accepted job offers (n = 4). Demographics of these 166 CICU-focused cardiologists were similar to the full cohort (Supplemental Table 2). The rate of burnout in the cardiologist subgroup was similar to those without certification in cardiology (36% vs 31%; *P* = 0.475). Burnout was again more common in women than in men in the cardiologist group, but this was no longer statistically significant (50% vs 32.8%; *P* = 0.25), whereas burnout rates remained statistically higher in later career stages (46.4% vs 22.4%; *P* = 0.01). The trends toward increased burnout in those physicians working with trainees (38.6% vs 25.8%; *P* = 0.18) and those not working at heart transplant centers (44.6% vs 30.6%; *P* = 0.07) also persisted but again did not reach statistical significance (Supplemental Table 3). There remained no significant difference in burnout among CICU-focused cardiologists working with advanced practice providers, those working in high vs low-intensity CICUs, those above/below the median number of ICU day and night shifts, above/below the median salary, or those with/without critical care medicine certification (Supplemental Table 4). Quartile analyses in the cardiologist subgroup were also similar to the overall sample, with lower burnout in older, later career stage CICU-focused cardiologists, and a trend toward less burnout in those working 48 to 112 total shifts annually compared to fewer or more (Supplemental Table 5).

## Discussion

In this survey analysis of over 200 CICU-focused physicians, we found that although the majority is satisfied with their current job, many continue to struggle with work-life balance and burnout. We identified a burnout rate of 35%, which did not significantly change when limiting the cohort to just those providers with cardiology board eligibility/certification. Burnout was more prevalent in women physicians as well as those later in their career, but interestingly, there was no clear relationship between burnout and number of ICU shifts (nights or days) or salary. These results may challenge the assumption that clinical load and financial compensation are primary drivers of burnout and stress the need to address other contributors. Overall, approximately one-third of the already small CICU workforce was considering a change in practice, with these individuals citing burnout as well as dissatisfaction with the work environment and institutional leadership as primary drivers.

High-intensity and emotionally demanding specialties such as emergency medicine, critical care medicine, and hospitalist medicine have historically been implicated as especially taxing on providers.^17^, ^18^, ^19^ Among critical care physicians specifically, cited burdens related to burnout include a unique exposure to patient pain and suffering (sometimes referred to as “compassions fatigue”) as well as the need to provide perceived futile care. Multiple publications have promoted the recognition of critical care burnout and recommended both individual and systematic changes to combat burnout.^20^, ^21^, ^22^ There has also been increased focus on physician well-being, psychological stress, and burnout among cardiologists. In the largest study to date, The ACC’s Professional Life Survey was analyzed for physician burnout, revealing a moderate rate of 27%, without significant differences across cardiology subspecialties or practice settings.^16^ More recent data, although including analyses during the COVID-19 pandemic, have identified burnout rates of 40%.^23^ There is increased awareness of the prevalence of mental health conditions in cardiologists, with 1/4 of all cardiologists reporting at least psychological distress, predicted by female gender, experiencing harassment or discrimination, age <55, and being divorced.^24^

We hypothesized that burnout would be high among CICU-focused physicians, given that the CICU represents a high stress environment with long hours and frequent emotional trauma more analogous to acute care practice environments than general cardiology practice. To this end, we identified a burnout rate of 35%, which is higher than that identified in other cardiology surveys including among all-comers (27%) and fellowship program directors (21%).^15^^,^^16^ Notably, our burnout rate was lower than that described in a workforce survey performed during the COVID-19 pandemic (40%) and in comparison to a recently published analysis of international interventional cardiologists (>60%).^23^^,^^25^ It is no surprise that burnout was higher during the peak challenges of COVID-19; however, the difference between our burnout rate and that identified in the interventional cardiology survey is likely attributable to vastly different burnout assessment tools. This highlights one of the main difficulties in burnout research—lack of a unified definition and measurement strategy.^26^

Similar to other cardiology and general physician burnout analyses, we found increased burnout among women providers.^6^^,^^15^^,^^16^ Drivers of increased burnout in women physicians are likely multifactorial with possible factors including higher workloads, fewer provided resources, less control over scheduling, as well as ongoing systemic cultural biases (such as lower compensation, exposure to harassment, and disproportionate responsibilities outside of work, such as childrearing and/or caregiving).^27^ Indeed, women cardiologists have recently been shown to experience more discrimination and harassment than their male colleagues, with these experiences linked to burnout, social avoidance, and consideration of leaving medicine.^28^ It is no surprise that women CICU-focused physicians face similar challenges to their colleagues, potentially heightened by the stressors of high intensity work and exposure to substantial patient morbidity and end of life care.

Our finding of increased burnout among later career physicians was perhaps less expected than that of the impact of gender. Some of the highest profile studies of physician burnout have indicated that burnout is highest in early career, when clinicians are less accustomed to their duties, perhaps less efficient with bureaucratic work, and more likely to be dealing with home challenges (such as parenting young children).^6^^,^^29^ However, there are also studies demonstrating that burnout and emotional exhaustion peak in midcareer, after clinicians have been in practice for 10 to 20 years.^16^^,^^30^ In the nascent field of CCC, relatively few physicians can likely be considered late career (>20 years in practice), with a substantial number of early and midcareer providers. Although early career faculty face unique challenges, the higher “later” career burnout observed in this study may indicate that the stressors of ICU care do not lessen with time, and that the associated emotional exhaustion accumulates as the career progresses.

Surprisingly, we did not discover a relationship between burnout and work expectations in terms of day or night shift counts; however, when total annual shifts were analyzed as quartiles, a trend emerged indicating less burnout associated with working 55 to 110 shifts. Burnout appeared to increase with both fewer (<55) and more (>110) shifts, potentially indicating that burnout is best prevented by working enough shifts to remain clinically sharp but not so many as to develop emotional exhaustion. We also did not identify a difference in burnout based on salary or between those with and without critical care medicine training, indicating that financial support and extra exposure to critical care medicine do not provide protection from burnout symptoms. Although we were able to identify that burnout was a large contributor (61%) to CICU-focused physicians’ intent to change practice, the present survey was designed as a general workforce survey and thus it did not go into detail about other potential contributing factors. There has been increasing recognition that cardiologists frequently battle mental health challenges as well as workplace mistreatment, the latter of which is associated with significant burnout.^24^^,^^31^ Future CICU workforce surveys should provide more detailed exploration of CICU-focused physician burnout, including examination of contributing factors that have been previously identified, such as nonclinical administrative burden, perceived lack of clinical autonomy, exposure to patient suffering, and workplace harassment. It is likely that CICU physicians face similar challenges to their non-CICU focused ICU colleagues and to this end, we can likely learn from and implement previously tested burnout mitigation strategies while still accumulating CICU specific data that may expose CICU-specific interventions. Although data are sparse, the burnout mitigation literature promotes addressing both individual and systems-level factors that contribute to burnout. Individual-based interventions include programs to build resiliency, foster mindfulness, and promote healthy living/combat fatigue; however, these have routinely been shown to have less impact than organizational interventions, primarily aimed to reduced workload, including overnight and weekend call burden.^32^, ^33^, ^34^, ^35^, ^36^, ^37^, ^38^ Understanding and counteracting CICU burnout early in the growth of CCC as a field should be prioritized, hopefully enabling sustained trainee interest and fostering lengthy and fulfilling careers.

It is important to note that the literature and discussions surrounding physician burnout are evolving, and the term burnout is falling out of favor. The reason for this is the pejorative implication that physician burnout is a personal failing of the individual, when the symptoms attributable to burnout typically result from the physician’s interaction with systematic problems within the healthcare environment. We have used the term “burnout” in our study due to its familiarity with most providers, recognizing that this space needs new terminology. We encourage providers who are suffering from symptoms that could be attributable to burnout or mental health conditions to take advantage of available support resources.

### Study Limitations

As with all voluntary survey studies, this analysis is subject to respondent bias and a potential lack of representativeness. We could not determine whether the presence of burnout influenced a decision regarding whether to participate in the survey and affected our observed burnout rates. Likewise, the anonymity of the survey could have affected individuals’ responses, which additionally could have varied over time with our survey being a single cross-sectional assessment. We used a few basic survey questions to assess job satisfaction and burnout and further dichotomized the presence of burnout for analysis. We recognize that more detailed assessment tools could have provided greater nuance or different points estimates and that burnout may change over time and warrants repeat assessment. Furthermore, we did not assess concurrent diagnosable mental health conditions and could not distinguish respondent-reported burnout from symptoms referable to such conditions. Finally, we performed exploratory burnout analyses to identify possible correlates of physician burnout in the CICU and foster future research. We did not account for multiple comparisons and thus associations must be interpreted with caution and warrant future confirmation.

## Conclusions

Burnout impacts over one-third of CICU-focused physicians. Burnout is more common in women and later career stages and is the leading reason cited by CICU-focused physicians that are considering practice change. The high rates of burnout we identified using a simplified dichotomized burnout screening tool are worrisome in the context of the burgeoning field of CCC and necessitate further research using more nuanced and validated burnout assessment tools. More data are needed regarding individual behaviors and institutional characteristics most associated with burnout, and how we can improve physician well-being to safeguard high value CICU care.

## Funding support and author disclosures

The views, information or content, and conclusions presented do not necessarily represent the official position or policy of, nor should any official endorsement be inferred on the part of, the Clinical Center, the National Institutes of Health, or the Department of Health and Human Services. Dr Barnett reports consulting for Abiomed and Abbott; and received research funding from Merck and Pfizer. Dr Brusca reports consulting for Johnson and Johnson; received research funding from 10.13039/100004331Johnson and Johnson; and serves as clinical trial enroller for Merck and Pfizer. Dr Lee receives honoraria from Getinge. Dr Thacil receives honoraria from Doximity. Dr Solomon receives research support from the 10.13039/100000002National Institutes of Health Clinical Center intramural research funds. All other authors have reported that they have no relationships relevant to the contents of this paper to disclose.
