# Supplementary material for: Physician Burnout and Work Satisfaction in the Cardiac Intensive Care Unit: Sponsored by the Critical Care Cardiology Section of the American College of Cardiology
Source: JACC Adv. 2026 Mar 25;5(3):102629. doi: 10.1016/j.jacadv.2026.102629 (PMC13352020; doi:10.1016/j.jacadv.2026.102629)
Supplement: Supplemental data [file mmc1.pdf]

Supplemental material

Supplemental Table 1: Quartile Analysis of Burnout in CICU-focused Physicians

	N-size	No Burnout	Burnout	P value*
Total	215	65.1%	34.9%	
**Career Stage**				**0.067**
0-3 years	43	83.7%	16.3%	
4-7 years	49	61.2%	38.8%	
8-16 years	45	57.8%	42.2%	
17 or more years	45	58.7%	42.2%	
Unknown	32	65.6%	34.4%	
**Age**				**0.093**
34-39 years old	49	81.6%	18.4%	
40-44 years old	41	63.4%	36.6%	
45-55 years old	47	57.4%	42.6%	
56 years and older	48	60.4%	39.6%	
Unknown	30	60.0%	40.0%	
**Medical and surgical CICU Beds**				**0.524**
0 to 10	7	57.1%	42.9%	
11 to 20	25	60.0%	40.0%	
More than 20	145	68.3%	31.7%	
Not sure/No answer	38	57.9%	42.1%	
**Total ICU Shifts Annually**				**0.030**
0 – 54	53	54.7%	45.3%	
55 – 110	56	80.4%	19.6%	
111 – 169	52	59.6%	40.4%	
More than 169	54	64.8%	35.2%	
**CICU Admissions Per Month**				**0.994**
0 – 100	62	66.1%	33.9%	
101 – 200	42	66.7%	33.3%	
201 – 250	18	61.1%	38.9%	
More than 250	53	64.2%	35.8%	
Not sure/No answer	40	65.0%	35.0%	
**Salary (U.S.)**	204			**0.489**
$100,000 - $324,999	39	76.9%	23.1%	
$325,000 - $374,999	32	59.4%	40.6%	
$375,000 - $474,999	56	62.5%	37.5%	
$475,000 and higher	58	63.8%	36.2%	
Refused/No answer	19	57.9%	42.1%	

*used chi-square analysis
Abbreviations: CICU, cardiac intensive care unit

Supplemental Table 2: Demographics for Respondents board eligible/certified in cardiovascular medicine and excluding all trainees (CICU cardiologists).

	**Count**	**Percent**
Total	N=166	100%
**Gender**		
Male	118	71.1%
Female	27	16.3%
Not reported	21	12.7%
**Age**		
31-40 years old	43	25.9%
41-50 years old	54	32.5%
51-60 yrs old	20	12.0%
61-70 years old	23	13.9%
71 years and older	8	4.8%
Not reported	18	10.8%
**Career Stage**		
In training	1	0.6%
1-7 years	59	35.5%
8-14 years	33	19.9%
15-21 years	15	9.0%
22 or more years	37	22.3%
Not reported	21	12.6%
**Location**		
United States	157	94.6%
Canada	9	5.4%

**Board Certification (multiple selections possible)**		
Cardiovascular disease	166	100%
Internal Medicine	115	69.3%
Critical care Medicine	76	45.8%
Echocardiography	62	37.3%
Advanced Heart Failure/Transplant	35	21.1%
Interventional Cardiology	27	16.3%
Electrophysiology	3	1.8%
**Employment Status**		
Currently in training, but have accepted a position	0	0%
Employed Part-time	5	3.0%
Employed Full-time	157	94.6%
Other	4	2.4%
**Practice Affiliation**		
University affiliated academic medical center	107	64.5%
Non-university associated academic medical center	28	16.9%
Community hospital	23	13.9%
VA or other government hospital	6	3.6%
Other, please specify	2	1.2%
**Academic Appointment/Faculty Rank**		
Instructor	4	2.4%
Assistant professor	71	42.8%
Associate professor	33	19.9%
Professor	27	16.3%

Other	1	0.6%
None, do not have an academic appointment	28	16.9%
Not reported	2	1.2%
**Race/Ethnicity**		
White	104	62.7%
Black/African American	5	3.0%
Hispanic/Latin	14	8.4%
Asian	32	19.3%
Native American/Alaskan Native	0	0%
Native Hawaiian/Pacific Islander	0	0%
Other	6	3.6%
Not reported	12	7.2%

Supplemental Table 3: CICU Cardiologist burnout as relates to demographics

	**N**	**No Burnout**	**Burnout**	**P value***
Total	163	63.8%	36.2%	
**Gender**				**0.25**
Male	116	67.2%	32.8%	
Female	26	50.0%	50.0%	
Not reported	21	61.9%	38.1%	
**Years in practice**				**0.01**
1-7 years	58	77.6%	22.4%	
8 or more years	84	53.6%	46.4%	

Not reported	21	66.7%	33.3%	
**Age**				**0.32**
31-50 years old	95	68.4%	31.6%	
51 years and older	50	56.0%	44.0%	
Not reported	18	61.1%	38.9%	
**Medical and surgical CICU Beds**				**0.36**
1 to 20	26	61.5%	38.5%	
More than 20	107	67.3%	32.7%	
Not reported	30	53.3%	46.7%	
**CICU Staffing Intensity#**				**0.37**
Traditional/Low-intensity	51	58.8%	41.2%	
High-intensity	78	62.8%	37.2%	
Not reported	34	73.5%	26.5%	
**Housestaff in the CICU**				**0.18**
No	31	74.2%	25.8%	
Yes	132	61.4%	38.6%	
**APPs in the CICU**				**0.63**
No	46	60.9%	39.1%	
Yes	117	65%	35%	
**Heart transplants performed**				**0.07**
No	65	55.4%	44.6%	
Yes	98	69.4%	30.6%	

*Chi-square; of the total 166 respondents, there were 3 that did not answer burnout questions.
Abbreviations: CICU, cardiac intensive care unit

Supplemental Table 4: CICU Cardiologist burnout as relates to number of ICU shifts, salary, days free of clinical service, and board certification in critical care medicine

	**N**	**No Burnout**	**Burnout**	**P value***
**ICU Shifts**				**0.687**
Median or below	86	62.4%	37.6%	
Above median	80	65.4%	34.6%	

**Current Salary – US only**				**0.449**
Below median	55	67.3%	32.7%	
Median or above	83	63.9%	36.1%	
No answer	16	50%	50%	
**Days free of clinical duty**				**0.225**
0-2 days	97	61.9%	38.1%	
3 or more days	35	71.4%	28.6%	
No answer/other	13	84.6%	15.4%	
**Board Certification**				**0.592**
Dual Critical Care/Cardiology	75	66.7%	33.3%	
Cardiology only	30	66.7%	33.3%	
Cardiology plus other subspecialty	58	58.6%	41.4%	

*Chi-square

Supplemental Table 5: Quartile Analysis of Burnout in CICU cardiologists

	**N-size**	**No Burnout**	**Burnout**	**P value***
Total	163	63.8%	36.2%	
**Career Stage**				**0.087**
0-4 years	38	78.9%	21.1%	
5-7 years	37	67.6%	32.4%	
8-16 years	31	48.4%	51.6%	
17 or more years	34	55.9%	44.1%	
Unknown	23	65.2%	34.8%	
**Age**				**0.013**
34-39 years old	31	87.1%	12.9%	
40-44 years old	40	65.0%	35.0%	
45-58 years old	36	58.3%	41.7%	

59 years and older	36	50.0%	50.0%	
Unknown	21	66.7%	33.3%	
**Medical and surgical CICU Beds**				**0.463**
0 to 10	6	50.0%	50.0%	
11 to 20	20	65.0%	35.0%	
More than 20	107	67.3%	32.7%	
Not sure/No answer	30	53.3%	46.7%	
**Total ICU Shifts Annually**				**0.342**
0 – 47	40	52.5%	47.5%	
48 – 112	45	71.1%	28.9%	
113 – 180	39	64.1%	35.9%	
More than 180	39	66.7%	33.3%	
**CICU Admissions Per Month**				**0.771**
0 – 100	44	70.5%	29.5%	
101 – 200	36	63.9%	35.1%	
201 – 250	13	61.5%	38.5%	
More than 250	41	63.4%	36.6%	
Not sure/No answer	29	55.2%	44.8%	
**Salary (U.S.)**	154			**0.456**
$100,000 - $324,999	32	75.0%	25.0%	
$325,000 - $374,999	23	56.5%	43.5%	
$375,000 - $474,999	45	62.2%	37.8%	
$475,000 and higher	38	65.8%	34.2%	
Refused/No answer	16	50.0%	50.0%	

*used chi-square analysis
Abbreviations: CICU, cardiac intensive care unit

Full survey:

2023 CCC Professional Assessment Survey

This research seeks to gather data regarding the training, certification and personal practice of physicians who practice in the field of critical care cardiology with the goal of understanding the current state and perspectives about the future direction of critical care cardiology physicians.

Qualifiers:
**Board eligible or board certified = eligible to be certified or certified by ABIM or similar organization
** shift= a block of time typically 8-12 hours of clinical responsibility, i.e. a week long block of ICU coverage= 7 day shifts**
** board certified/board eligible= according to certification rules of ABIM (or similar organization)**

On behalf of the American College of Cardiology, thank you for your participation in this important research. This survey will take less than 8-10 minutes to complete.

When you have completed all of the questions, be sure to click on the "Submit" button. Once you submit your answers, you will not be able to return to this survey to make changes. If you need to exit the survey before you are finished, please select the "Save" button. Your responses will be saved and you can resume taking the survey from the point at which you stopped.

Thank you very much for your time. We look forward to your feedback!

To begin...

1. Please select all of the following for which you are currently or previously ABIM (or similar organization) board eligible or board certified: *answer required*
    - ☐ Internal medicine
    - ☐ Anesthesia
    - ☐ Emergency medicine
    - ☐ Surgery
    - ☐ Cardiovascular disease
    - ☐ Critical care medicine
    - ☐ Advanced heart failure and transplant cardiology
    - ☐ Interventional cardiology
    - ☐ Electrophysiology
    - ☐ Echocardiography
    - ☐ Neurocritical care
    - ☐ Pulmonary medicine
    - ☐ Other (write in)
    - ☐ Not board certified / board eligible in any field of medicine – THANK AND TERMINATE

2. What is your employment status? *answer required*
    - o Currently in training and have not accepted a position THANK AND TERMINATE
    - o Currently in training, but have accepted a position
    - o Employed Part-time
    - o Employed Full-time
    - o Other, please specify: ________

3. What best describes the affiliation of your primary practice facility? *answer required*
    a. University affiliated academic medical center
    b. Non-university associated academic medical center
    c. Community hospital
    d. VA or other government hospital
    e. Other, please specify

f. Not in practice – THANK AND TERMINATE

4. If you have an academic appointment, what is your faculty rank?
   - o Instructor
   - o Assistant professor
   - o Associate professor
   - o Professor
   - o Other, please specify:
   - o None, do not have an academic appointment

5. Please indicate your race/ethnicity (check all that apply)
   - ☐ White
   - ☐ Black/African American
   - ☐ Hispanic/Latin
   - ☐ Asian
   - ☐ Native American/Alaskan Native
   - ☐ Native Hawaiian/Pacific Islander
   - ☐ Other, specify: __________
   - ☐ Do not wish to disclose

6. How often do you round in the medical or surgical CICU as a primary managing clinician (e.g. you are in charge of primary decisions rather than rounding as a consultant)? *answer required*
   - o More than 20 weeks/year
   - o 10 weeks to 20 weeks/year
   - o 6 weeks to less than 10 weeks/year
   - o 2 weeks to less than 6 weeks/year
   - o Less than 2 weeks/year
   - o Do not round as a CICU cardiologist – THANK AND TERMINATE

Turning to the organization of care delivery at your institution

7. Approximately how many total hospital beds and medical and surgical CICU beds are there in your primary institution?

Total Hospital Beds	Medical and surgical CICU Beds
o None<br>o 1 to 50<br>o 51 to 100<br>o 100 to 200<br>o 201 to 300<br>o 301 to 400<br>o 401 to 500<br>o More than 500<br>o Not sure	o None<br>o 1 to 5<br>o 6 to 10<br>o 11 to 20<br>o 21 to 30<br>o 31 to 40<br>o 41 to 60<br>o More than 60<br>o Not sure

8. Approximately, how many medical and surgical CICU patients are treated <u>each month</u> in your institution?
   - o None
   - o 1 to 25
   - o 26 to 50
   - o 51 to 75
   - o 76 to 100
   - o 101 to 150
   - o 151 to 200
   - o 201 to 250
   - o More than 250
   - o Not sure

9. What best describes the cardiovascular clinical capabilities of your primary institution? Please select one response.
   - o Manage all cardiovascular diagnoses
   - o Manage most cardiovascular diagnoses
   - o Manage many cardiovascular diagnoses
   - o Manage some cardiovascular diagnoses
   - o Manage few cardiovascular diagnoses
   - o Initial diagnosis and stabilization of common cardiovascular conditions, transfer to another facility for definitive care
   - o Other, please specify: ______-
   - o Not sure

10. What best describes the cardiac transplant and MCS capabilities of your primary institution? (Check all that apply)
    - Perform heart transplant
    - Implant and manage durable MCS (durable LVAD)
    - Initiate and manage V-A ECMO (tandem heart, centrimag, cardiohelp)
    - Initiate and manage catheter based temporary LVAD (impella)
    - Initiate and manage IABP
    - Initiate V-A ECMO (tandem heart, centrimag, cardiohelp)
    - Initiate catheter based temporary LVAD (impella)
    - Initiate IABP
    - No transplant, durable or temporary MCS or IABP
    - Other, please specify: _____-
    - Not sure

11. What best describes the organization of care for medical CICU patients and surgical CICU patients at your primary facility? (Check all that apply)

Medical CICU patients	Surgical CICU patients
☐ Mixed ICU for all critically ill patients (including both medical and surgical)	☐ Mixed ICU for all critically ill patients (including both medical and surgical)

☐ Mixed ICU for medical and cardiac critically ill patients<br>☐ ICU dedicated to medical cardiac critically ill patients only<br>☐ Other<br>☐ Not sure	☐ Mixed ICU for medical and cardiac critically ill patients<br>☐ ICU dedicated to medical cardiac critically ill patients only<br>☐ Other<br>☐ Not sure

12. Please select the options which best describe the staffing model for each scenario at your primary facility. Please select all that apply.

	Medical CICU patients	Overnight coverage of medical CICU patients	Surgical CICU patients	Overnight coverage of surgical CICU patients
Any hospital credentialed cardiologist can manage a patient they admit to the CICU.	☐	☐	☐	☐
A critical care medicine board eligible/certified physician is available for consultation.	☐	☐	☐	☐
A critical care medicine board eligible/certified physician manages or co-manages patients as the primary provider.	☐	☐	☐	☐
A board eligible/certified critical care medicine and cardiology physician manages patients as the primary provider.	☐	☐	☐	☐
Trainees including residents or fellows participate in the management of patients.	☐	☐	☐	☐
Advanced practice providers participate in the management of patients.	☐	☐	☐	☐
A dual board eligible/certified critical care medicine and cardiology physician is on home call overnight	☐	☐	☐	☐ 13
A physician is not in house but is available by telephone	☐	☐	☐	☐
A non-critical care medicine certified physician is in house overnight	☐	☐	☐	☐
A critical care medicine certified physician is in house overnight	☐	☐	☐	☐
A dual board eligible/certified critical care medicine and cardiology physician is in house overnight	☐	☐	☐	☐
Other	☐	☐	☐	☐
Not sure	☐	☐	☐	☐

Now thinking about personal practice …

13. Approximately how many shifts per year do you spend doing each of the following activities by type of shift?

** shift= a block of time typically 8-12 hours of clinical responsibility, i.e. if you are assigned to 7 days of ICU coverage= 7 day shifts**

	Daytime shifts	IN HOUSE overnight Shifts	AT HOME overnight shifts
Medical CICU			
Surgical CICU			
Mixed medical and surgical CICU			
Any other ICU not reported above			

14. How many consecutive CICU shifts do you typically work?
   - o None
   - o 1
   - o 2
   - o 3
   - o 4
   - o 5
   - o 6
   - o 7
   - o 8 or more

[PROGRAMMER NOTE: ASK Q.15, IF Q.14= 1 or more consecutive ICU shifts]

15. Following the INSERT NUMBER FROM Q14 consecutive CICU shifts you work, how many days do you typically have free from all direct patient care duties?
   - o 0
   - o 1-2
   - o 3-5
   - o 6-7
   - o >7
   - o Other, please specify:

16. What procedures do you personally perform? Please select all that apply.
   - Intubation
   - Bronchoscopy
   - Chest tube insertion
   - Advanced bronchoscopy (cryotherapy, bronchial blockade)
   - Vascular access (arterial lines, central lines, PA catheters, hemodialysis lines)
   - Insertion of IABP
   - Insertion of ECMO
   - Insertion of percutaneous VAD (Impella®, TandemHeart®)
   - Temporary peacemaker insertion
   - Pericardiocentesis
   - Paracentesis
   - Thoracentesis
   - Transesophageal echo
   - Electrical cardioversion
   - CRRT
   - None of these

17. About how many weeks a year do you spend on the following non-ICU activities?

	1 week or less	2-10 weeks	11 – 20 weeks	21 – 30 weeks	31 – 40 weeks	41 or more weeks	N/A
Outpatient clinic	o	o	o	o	o	o	o
Echocardiograph	o	o	o	o	o	o	o
Nuclear/CT/MRI	o	o	o	o	o	o	o
Interventional cardiology	o	o	o	o	o	o	o
Cardiology Consult Service	o	o	o	o	o	o	o
Electrophysiology	o	o	o	o	o	o	o
Advanced heart failure/transplant and pulmonary hypertension	o	o	o	o	o	o	o
Inpatient cardiology service, non-CICU	o	o	o	o	o	o	o
Research	o	o	o	o	o	o	o
Administrative	o	o	o	o	o	o	o

18. In your opinion, what is the maximum number of IN-HOUSE shifts (total daytime AND nighttime) per year that a physician must work in a CICU ~~to maintain critical care skills~~ without contributing to burn out? _____

19. Based on how you would define burnout for yourself, how would you rate your overall level of burnout?
    - o I enjoy my work. I have no symptoms of burnout.
    - o Occasionally I am under stress, and I don't always have as much energy as I once did, but I don't feel burned out.
    - o I am definitely burning out and have one or more symptoms of burnout, such as physical or emotional exhaustion
    - o The symptoms of burnout that I'm experiencing won't go away. I think about frustration at work a lot.
    - o I feel completely burned out and often wonder if I can go on. I am at the point where I may need some changes or may need to seek some sort of help.

20. Please rate your satisfaction on the following: 1=Not at all satisfied <--- > 5 = Extremely satisfied / Not applicable
    a) Work-life balance
    b) Satisfaction with current job
    c) Required work performed at home after your shifts
    d) Work-related challenges regarding childcare/family duties

21. At your primary institution, approximately how many CICU shifts (about 12 hours per shift) per year constitutes 100% clinical FTE?
    - <50
    - 50-70
    - 71-90
    - 91-110
    - 111-130
    - 131-150
    - 151-170
    - 171-190
    - 191-210
    - 211-230
    - 231-250
    - 251-270
    - 271-290
    - >290
    - Other, please specify: _______
    - Not sure

22. At your primary institution, what IS the approximate compensation for 100% clinical FTE composed entirely of CICU time, including all bonus payments, excluding benefits? In your opinion what should be the appropriate compensation for 100% clinical FTE composed entirely of CICU time, including all bonus payments, excluding benefits?

Current Compensation	Desired Compensation
○ I am in training	○ I am in training
○ <100 K	○ <100 K
○ 100-150K	○ 100-150K
○ 150-200K	○ 150-200K
○ 200-250K	○ 200-250K
○ 250-300K	○ 250-300K
○ 300-350K	○ 300-350K
○ 350-400K	○ 350-400K
○ 400-450K	○ 400-450K
○ 450-500K	○ 450-500K
○ 500-550K	○ 500-550K
○ 550-600K	○ 550-600K
○ 600-650K	○ 600-650K
○ 650-700K	○ 650-700K
○ >700K	○ >700K

23. During the next 12 months, how likely are you to do the following: (select all that apply)

- ☐ I plan to reduce my clinical work hours
- ☐ I plan to leave my current practice setting/employer
- ☐ I plan to retire early
- ☐ None of the above

[PROGRAMMER NOTE: IF Q.23=Yes, PLEASE ASK Q.23A]
23A. Why do you plan on changing your practice in the next 12 months? Please select all that apply.

- Inadequate compensation
- Overworked/burnout
- Dissatisfaction with work environment
- Dissatisfaction with leadership
- Interested in pursuing new opportunities at another organization
- Planning to retire
- Other, please specify: ________
- Not sure

24. Lastly, please feel free to share any additional comments about the field of critical care cardiology.

____

____

____

Thank you so much for your valuable insights. If you would like your name to be entered into a drawing to win one of four $250 Amazon cards, please click submit and you will be taken to a page to provide your contact information.

On end page:

Would you like to:

- ☐ Join/Participate in ACC's Critical Care Cardiology Section – no cost
- ☐ Enter into a drawing to win one of four $250 Amazon cards
- ☐ Both of the above
- ☐ None

IF Join section and/or Enter drawing, ask:

Please provide your name and email address:
Name:
Email address:

DEMOGRAPHICS FROM ACC DATABASE:

Practice location / state / region
Training end year
Gender
